# Altered Long Non-coding RNAs Involved in Immunological Regulation and Associated with Choroidal Neovascularization in Mice

**DOI:** 10.7150/ijms.37804

**Published:** 2020-01-16

**Authors:** Liwei Zhang, Huilan Zeng, Jiang-Hui Wang, Han Zhao, Boxiang Zhang, Jingling Zou, Shigeo Yoshida, Yedi Zhou

**Affiliations:** 1 Department of Ophthalmology, The Second Xiangya Hospital, Central South University, Changsha, Hunan, China; 2 Hunan Clinical Research Center of Ophthalmic Disease, Changsha, Hunan, China; 3 Centre for Eye Research Australia, Royal Victorian Eye and Ear Hospital, East Melbourne, Victoria, Australia; 4 Ophthalmology, Department of Surgery, University of Melbourne, East Melbourne, Victoria, Australia; 5 Department of Ophthalmology, Kurume University School of Medicine, Kurume, Fukuoka, Japan

**Keywords:** long non-coding RNA, choroidal neovascularization, angiogenesis, age-related macular degeneration, immunological regulation

## Abstract

Choroidal neovascularization (CNV) is a severe complication of the wet form of age-related macular degeneration (AMD). Long non-coding RNAs (lncRNAs) have been implicated in the pathogenesis of different ocular neovascular diseases. To identify the function and therapeutic potential of lncRNAs in CNV, we assessed lncRNAs and mRNA expression profile in a mouse model of laser-induced CNV by microarray analysis. The results of altered lncRNAs were validated by qRT-PCR. Bioinformatics analyses, including Gene Ontology (GO) analysis and Kyoto Encyclopedia of Genes and Genomes (KEGG) pathway analysis, were performed to clarify the potential biological functions and signaling pathways with which altered genes are most closely related. Moreover, to identify the interaction of lncRNAs and mRNAs, we constructed a coding-non-coding gene co-expression (CNC) network. By microarray analysis, we identified 716 altered lncRNAs and 821 altered mRNAs in CNV mice compared to controls. A CNC network profile based on 7 validated altered lncRNAs (uc009ewo.1, AK148935, uc029sdr.1, ENSMUST00000132340, AK030988, uc007mds.1, ENSMUST00000180519) as well as 282 interacted and altered mRNAs, and were connected by 713 edges. GO and KEGG analyses suggested that altered mRNAs, as well as those lncRNA-interacted mRNAs were enriched in immune system process and chemokine signaling pathway. Thus, lncRNAs are significantly altered in this mouse model of CNV and are involved in immunological regulation, suggesting that lncRNAs may play a critical role in the pathogenesis of CNV. Thus, dysregulated lncRNAs and their target genes might be promising therapeutic targets to suppress CNV in AMD.

## Introduction

Age-related macular degeneration (AMD) is one of the major causes of visual impairments and blindness in developed regions 1. Choroidal neovascularization (CNV) is a severe complication of the neovascular or wet AMD 2. It is well known that vascular endothelial growth factor (VEGF) plays key roles in the pathogenesis of neovascular AMD 3, 4. Numerous studies have demonstrated that anti-VEGF therapy is effective and safe for patients with CNV due to AMD for which it has been widely used in clinics 5, 6. However, recent studies suggest that some VEGF neutralizing proteins are not effective in some patients 7, 8. There is increasing evidence that a number of factors may be responsible for non-responses including tolerance or tachyphylaxis (a term refers to the sudden decrease in response to a drug after its administration) of anti-VEGF therapies 9, 10. Therefore, there is an urgent need for the mechanistic understanding of CNV, to explore novel therapeutic targets for early intervention of CNV, which would provide potential alternatives to anti-VEGF therapies.

Long non-coding RNAs (lncRNAs) is a subtype of non-coding RNAs with the transcripts of more than 200 nucleotides 11. Although lncRNAs have no protein-coding capacity, they may regulate physiological functions as well as pathological processes in many diseases 12-15. In particular, the activation of lncRNAs might regulate the expression of protein-coding genes through sophisticated mechanisms in ocular disorders 16-19. It has been reported that lncRNAs have differential expression profiles in a mouse model of ischemia-induced retinal neovascularization^20^. The role of lncRNAs in AMD has also been investigated in a number of studies. A study showed that lncRNAs are differentially expressed in RPE/choroid samples in patients with early AMD compared to controls and might be involved in important regulative functions 21. Among altered lncRNAs in patients with early AMD, RP11-234O6.2 has been demonstrated as having protective effects in the aging RPE model 21. A few *in vitro* studies investigated the roles of several certain lncRNAs (such as ZNF503-AS1 and BANCR) in retinal pigment epithelium (RPE) cells 22, 23. Nevertheless, it still remains unclear that the expression profiles, targets, and effects of lncRNAs and their contribution to the pathogenesis of CNV as well as neovascular AMD.

Laser-induced CNV is a well-established mouse model to investigate the pathogenesis of CNV and neovascular AMD 24. Previous studies using the CNV mouse model to examine the role of VEGF and many other molecules 25-27. We recently identified altered microRNAs and tRNA-derived small RNAs in the laser-induced CNV model 28.

In the present study, microarray analyses were applied to clarify the expression profiles of lncRNAs and mRNAs in the CNV mouse model. In addition, bioinformatics analyses, including gene ontology (GO) analysis and Kyoto Encyclopedia of Genes and Genomes (KEGG) analysis, were conducted to explore the related biological functions and potential signaling pathways of altered genes. Moreover, a coding-non-coding gene co-expression (CNC) network was established to investigate the correlation of differentially expressed lncRNAs and mRNAs, and to further predict the possible roles of differentially expressed lncRNAs in CNV.

## Materials and Methods

### Animals

C57BL/6J mice (male, aged 7-8 weeks old) (SJA Laboratory Animal Co., Ltd., Hunan, China) were used for experiments in accordance to the ARVO Statement for the Use of Animals in Ophthalmic and Vision Research. The procedures were proved by the Institutional Animal Care and Use Committee of Central South University.

### Laser-induced CNV mouse model

As previously described, the CNV mouse model was induced by laser photocoagulation 27-29 with some modification. In brief, the photocoagulation was performed with 25 spots in each eye using a 532-nm diode laser (100 mW, 0.1 s duration, 50 μm). On day 7 after laser photocoagulation treatment, the eyes were enucleated. Age-matched C57BL/6J mice without the treatment were used as controls.

### LncRNA and mRNA microarray analysis

Tissues of RPE-choroid-sclera complexes were collected from 4 eyes to be pooled as one sample, and a total of 6 samples (3 CNV samples and 3 control samples) were analyzed. Total RNA was isolated by using Trizol RNA extraction kit (Invitrogen, Carlsbad, CA, USA). The microarray analysis was performed by mouse lncRNA Microarray V3.0 (Arraystar, Rockville, MD, USA) as described 20. The raw data of microarray has been uploaded to the Gene Expression Omnibus database (http://www.ncbi.nlm.nih.gov/geo/) for public access with the accession number GSE129743. Altered lncRNAs and mRNAs expression were identified by a Volcano Plot filtering [fold change (FC) ≥1.5] and P<0.05.

### Quantitative real-time reverse transcription-polymerase chain reaction (qRT-PCR)

The validation of lncRNA and mRNA microarray results was further processed by qRT-PCR 30. RNA was transcribed into cDNA utilizing SuperScript Ⅲ Reverse Transcriptase (Invitrogen) following instructions of the manufacturer by Gene Amp PCR System 9700 (Applied Biosystems, Foster City, CA, USA). The qRT-PCR was performed by the ViiA 7 RT PCR System (Applied Biosystems) with the 2 × PCR Master Mix (Arraystar). LncRNAs' relative expression levels were normalized with GAPDH 31, 32. Primer used for qRT-PCR are shown in Table 1. P<0.05 was considered statistically significant differences.

### GO and KEGG pathway analyses

GO analysis (http://www.geneontology.org/) and KEGG pathway analysis (http://www.genome.jp/kegg/) were used to investigate the potential biological functions and significant pathways of the altered mRNAs or CNC-associated mRNAs.

### Construction of lncRNA-mRNA co-expression network

According to validated altered lncRNAs and their related mRNAs, we established a CNC network profile to explore the relationship between lncRNAs and mRNAs. We selected Pearson correlation coefficients (PCCs)≥ 0.97 to construct the network utilizing Cytoscape version 2.8.1 software (The Cytoscape Consortium, San Diego, CA, USA) based on the PCCs of correlation analysis of lncRNA and mRNA.

## Results

### Expression profiles of lncRNA and mRNA in CNV mice

**Figure 1:**
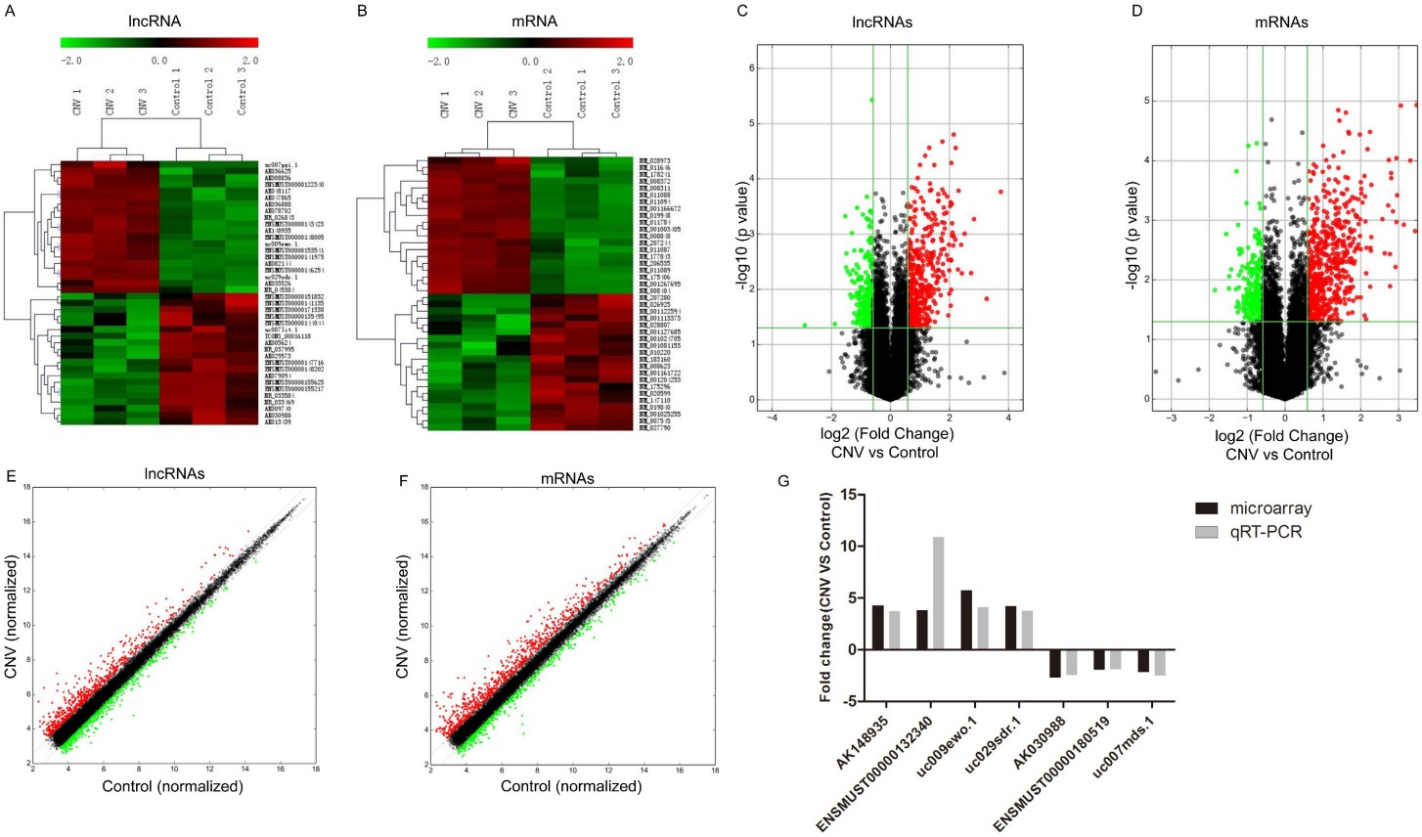
** Altered expressions of lncRNAs and mRNAs between CNV group and control group by microarray.** Heat map and hierarchical clustering analysis of the top 20 up- and downregulated lncRNAs (A) and mRNAs (B) between CNV and control samples. The top column represents lncRNA and mRNA relative expression varies according to the color scale. The volcano plot presents all identified lncRNA (C) and mRNA (D) expression variation between the CNV and control samples. The horizontal green line shows the default 1.5-fold change and the vertical green line represents a P-value of 0.05. The red and green plots represent up- and downregulated RNAs with FC≥1.5 and P<0.05. The scatter plot presents all identified lncRNA (E) and mRNA (F) expression variation between the CNV and control samples. The x-axis and y-axis values are each sample's normalized values (log2 transformed). The gray line shows the default 1.5-fold changes. The red and green plots represent altered RNAs with FC≥1.5. The qRT-PCR validation of seven randomly selected lncRNAs: AK148935, ENSMUST00000132340, uc009ewo.1, uc029sdr.1, AK030988, ENSMUST00000180519, uc007mds.1 (G).

To assess the expressions of lncRNAs in CNV compared to control mice, microarray analysis was conducted in the collected RPE-choroid-sclera complexes. Our results showed that 716 lncRNAs were significantly altered in CNV mice compared to control mice: 442 were upregulated and 274 were downregulated (FC≥1.5, P<0.05). The top 20 up- and downregulated lncRNAs are listed in Table 2-3. Among the differentially expressed lncRNA transcripts, AK036888 tops among upregulated lncRNAs with an FC of 13.38, whereas ENSMUST00000135495 tops among downregulated lncRNAs with an FC of 7.47. The clustering analysis demonstrated the relevance of lncRNA expression patterns in CNV and control samples by showing the top 20 up- and downregulated lncRNAs (Fig. 1A). The variation of lncRNA expression between CNV and control mice is shown in a volcano plot (Fig. 1C) and a scatter plot (Fig. 1E).

We also identified that 821 mRNAs were significantly altered in CNV mice compared to control mice: 588 were upregulated and 233 were downregulated (FC≥1.5 , P<0.05). The top 20 significantly altered mRNA are shown in Table 4-5. Among those upregulated mRNAs, Aplnr (NM_011784) tops with an FC of 11.11, while expression of Prpmp5 (NM_001024705) tops among downregulated genes with FC of 3.61. The heatmap plot revealed the clustering analysis among mRNA expression patterns by presenting the top 20 up- and downregulated mRNAs in the collected samples (Fig. 1B). A volcano plot (Fig. 1D) and a scatter plot (Fig. 1F) showed the variation of mRNA expression between CNV and control mice.

### Validation of the microarray data of lncRNAs by qRT-PCR

To validate the accuracy and reliability of the microarray profiling data, seven lncRNAs (uc009ewo.1, AK148935, uc029sdr.1, ENSMUST00000132340, AK030988, uc007mds.1, ENSMUST00000180519) were randomly selected for qRT-PCR. The qRT-PCR results are consistent with the microarray analyses where expression of lncRNAs AK148935, ENSMUST00000132340, uc009ewo.1 and uc029sdr.1 were significantly upregulated and expression of lncRNAs AK030988, ENSMUST00000180519 and uc007mds.1 were significantly downregulated in CNV mice compared to control mice (Fig. 1G).

### GO and KEGG pathway analyses of altered genes

**Figure 2:**
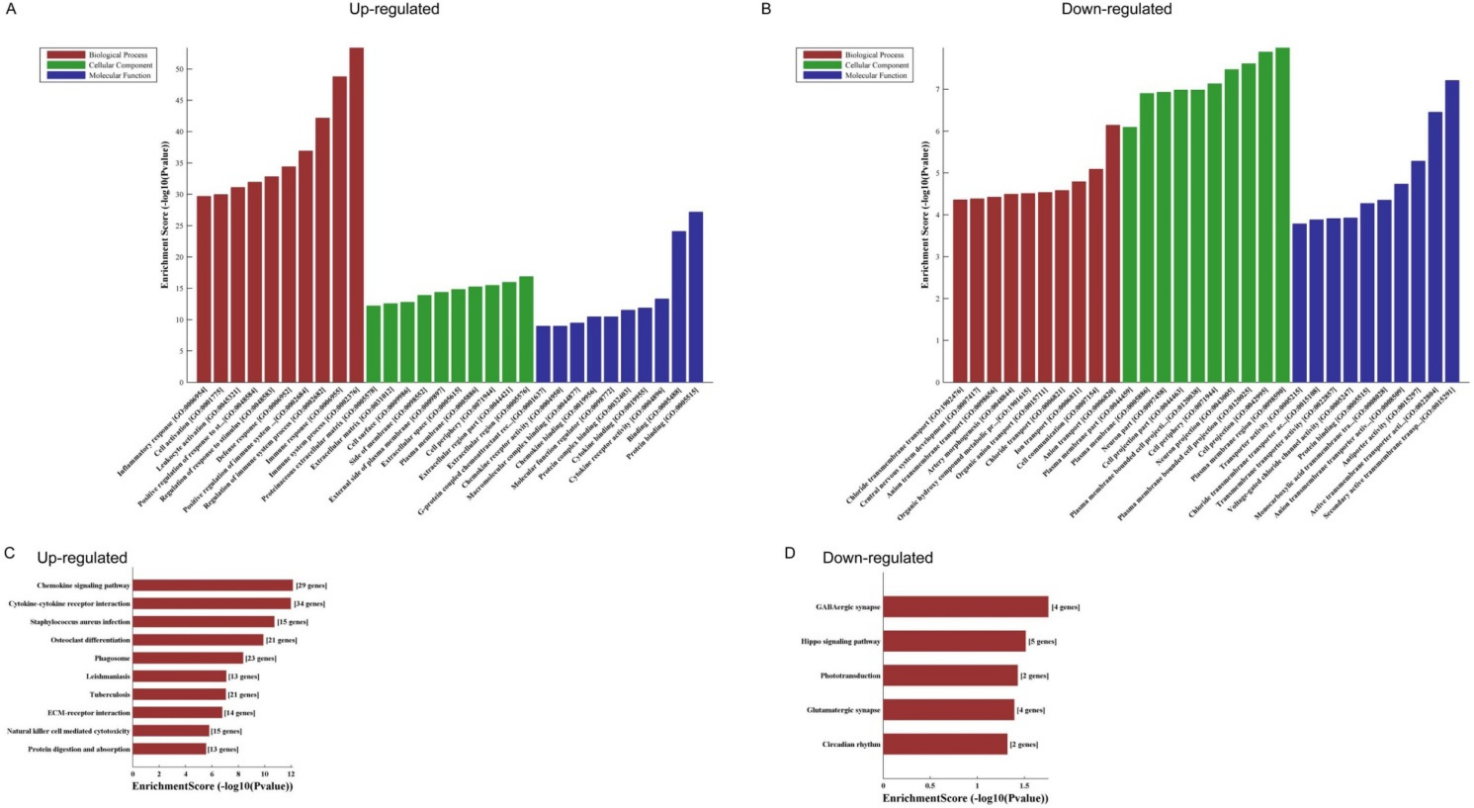
** The GO and KEGG analyses of altered mRNAs.** The GO analyses of up- (A) and downregulated (B) mRNAs. KEGG pathway analysis of altered mRNAs indicated: the top 10 significant enriched pathways of the upregulated genes (C), and the top 5 significant enriched pathways of the downregulated genes (D).

GO analyses of the 821 altered mRNAs showed that upregulated genes were enriched in immune system process (ontology: biological process, GO: 0002376), extracellular region (ontology: cellular component, GO: 0005576) and protein binding (ontology: molecular function, GO: 0005515) (Fig. 2A); while downregulated genes were enriched in anion transport (ontology: biological process, GO:0006820), plasma membrane region (ontology: cellular component, GO: 0098590) and secondary active transmembrane transporter activity (ontology: molecular function, GO: 0015291) (Fig. 2B).

KEGG pathway analysis of those differentially expressed mRNAs found that the upregulated genes were enriched in chemokine signaling pathway, cytokine-cytokine receptor interaction and *staphylococcus aureus* infection (Fig. 2C), while the downregulated genes were enriched in GABAergic synapse, Hippo signaling pathway and phototransduction (Fig. 2D).

### CNC networks with GO and KEGG analyses

**Figure 3:**
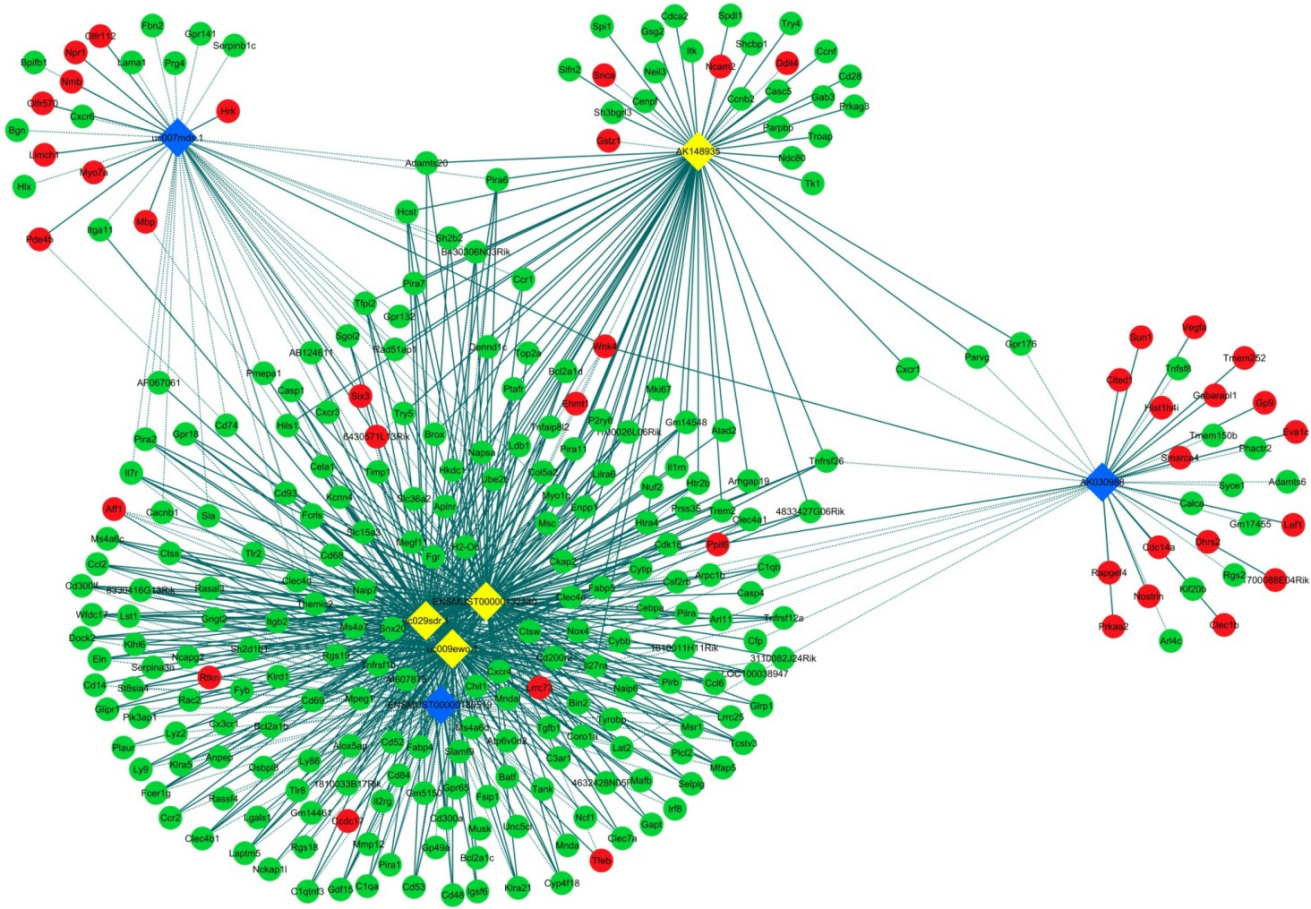
** CNC networks by validated lncRNAs.** The co-expression network profiles of lncRNAs and mRNAs based on 7 validated lncRNAs and correlated mRNAs which were differentially expressed. This co-expression network composed of 713 edges and 289 nodes. The diamond nodes represent lncRNAs, in which yellow denotes upregulated lncRNAs and blue denotes downregulated lncRNAs. The round nodes represent mRNAs, in which green denotes upregulated mRNAs and red denotes downregulated mRNAs. Continuous and dotted lines indicate positive and negative interactions between lncRNAs and mRNAs, respectively.

**Figure 4:**
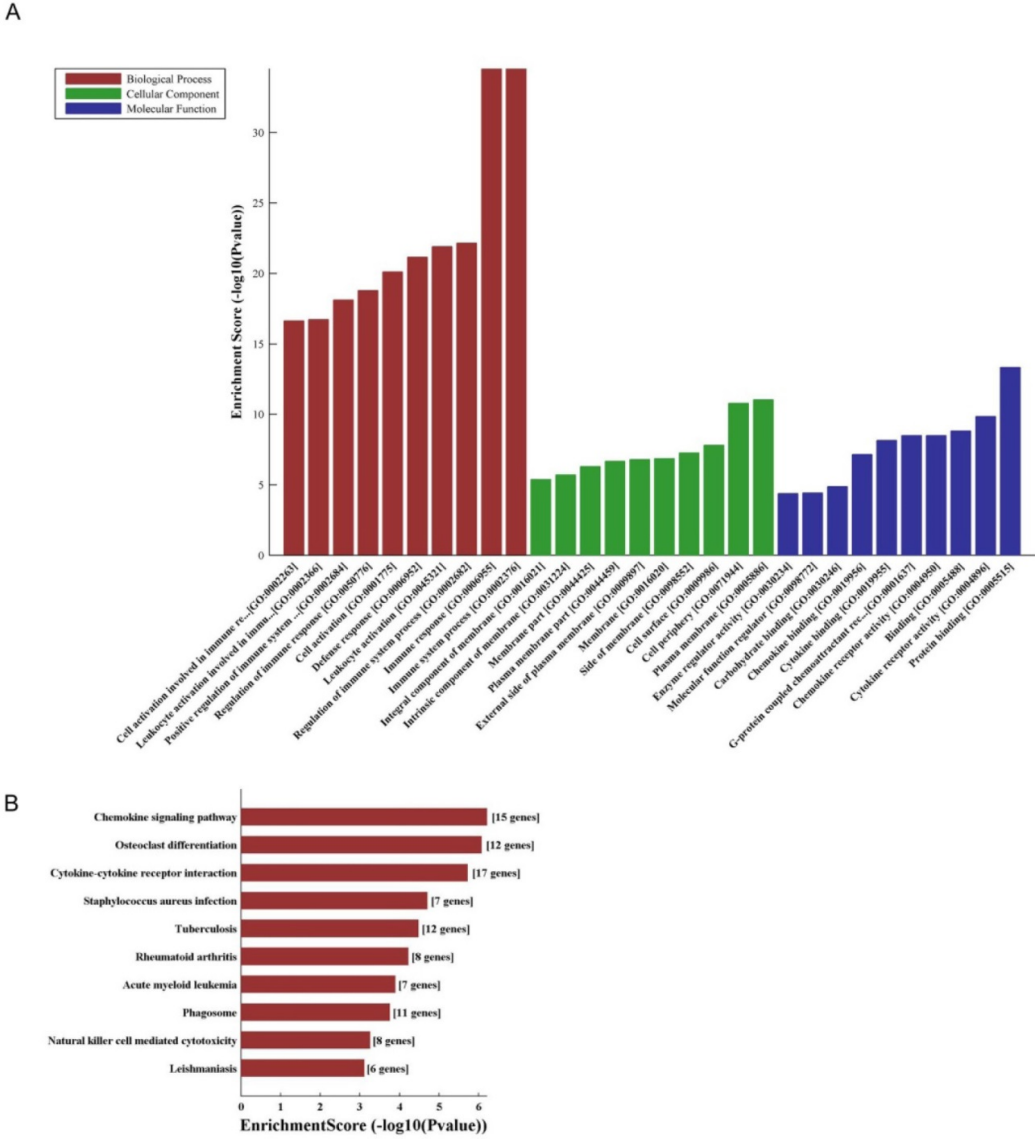
** The GO and KEGG analyses of interacted and altered mRNAs in CNC networks.** GO analysis of the CNC-associated altered mRNAs (A). KEGG pathway analysis of the CNC-associated altered mRNAs (B).

LncRNA and mRNA co-expression network was established based on 7 validated altered lncRNAs as well as 282 interacted mRNAs which were differentially expressed. The CNC network was composed of 289 nodes and 713 edges. There were 512 positive and 201 negative interactions within the network (Fig. 3). The lncRNA uc009ewo.1 is correlated with 138 mRNAs, ENSMUST00000132340 is correlated with 132 mRNAs, uc029sdr.1 is correlated with 152 mRNAs, ENSMUST00000180519 is correlated with 127 mRNAs, AK148935 is correlated with 76 mRNAs, while uc007mds.1 and AK030988 are correlated with 49 and 39 mRNAs respectively. According to the networks, the most relevant mRNAs are B430306N03Rik, C-type lectin receptor 4e (Clec4e) and paired immunoglobulin-like receptor A6 (Pira6), all correlated with 6 lncRNAs. To predict the functions of the lncRNAs, GO analyses and KEGG pathway analyses of those interacted mRNAs which were differentially expressed were conducted based on the results of the co-expression network. GO analysis showed that the most enriched GO terms of the targeted genes were immune system process (ontology: biological process, GO: 0002376), plasma membrane (ontology: cellular component, GO: 0005886) and protein binding (ontology: molecular function, GO: 0005515) (Fig. 4A). KEGG pathway analysis showed those targeted mRNAs were enriched in chemokine signaling pathway, osteoclast differentiation and cytokine-cytokine receptor interaction (Fig. 4B).

## Discussion

Despite several studies reported important roles of particular lncRNAs such as ZNF503-AS1 and BANCR in RPE cells through *in vitro* studies 22, 23, none of them have investigated the role of lncRNAs in CNV. The present study profiled lncRNA and mRNA expression in the mouse model of laser-induced CNV by integrated microarray analysis. We identified 821 significantly altered mRNAs (588 upregulated; 233 downregulated) and 716 differentially expressed lncRNAs (442 upregulated; 274 downregulated) in the RPE-choroid-sclera complexes from CNV mice compared to control mice. Validation of seven randomly chosen lncRNAs by qRT-PCR further confirmed the reliability of microarray analysis. The profiles of the lncRNAs and mRNAs in CNV mice provide novel insights into our understanding of the pathogenesis of CNV.

A number of studies have reported that different cytokines, such as IL-10, IL-12, IFN-γ, IL-17, IL-18 and IL-33, may play a role in the pathogenesis of CNV 33-37. The pro- and anti-angiogenic effects of the cytokines relevant to Th1, Th2 and Th17 cells, participate in a complicated immunological network 38. It has been reported that the number of macrophages increased significantly after laser photocoagulation 24, and M1-M2 polarization of macrophages have diverse distributions and functions in laser-induced CNV as well as in wet AMD 39-41. Pro-inflammatory M1 macrophages increased in the site of CNVs 39, suggesting that inflammation might play a key role in the pathogenesis of CNV and AMD. Our GO analyses showed that the most upregulated genes participate in immune system process, whereas the downregulated genes participate in plasma membrane region. Moreover, the KEGG pathway analysis revealed that the functions of altered genes are enriched in the chemokine signaling pathway, cytokine-cytokine receptor interaction and the GABAergic synapse. Similarly, GO and KEGG pathway analyses of the lncRNA-interacted mRNAs showed that the most enriched GO terms are associated with immune system process and immune response, and the most enriched KEGG pathway is also chemokine signaling pathway. These analyses demonstrated that CNV might be mainly immune-regulated by cytokines and chemokines.

Macrophage inducible Ca2+-dependent lectin receptor (Mincle) is a member of the C-type lectin family of immune receptors, which encoded by the gene of Clec4e, and Clec4e is also a type 2 transmembrane receptor 42. As the CNC network analysis showed, Clec4e is a significantly altered gene correlated with 6 validated lncRNAs. *Lv et al.* reported that clec4e is induced specifically on M1 macrophages, and it plays an essential role in maintaining the phenotype of M1 macrophage in acute renal inflammation 43. Another study showed that that Clec4e enhanced proinflammatory phenotype of macrophages through activation of the unfolded protein response 44. Our previous study demonstrated that M1 macrophages mainly distribute around the site of CNVs, and inflammatory M1 macrophage-associated cytokines increased in RPE and choroid in laser-induced CNV mouse model 39. Thus Clec4e, as well as macrophage polarization are possibly involved in mRNA-lncRNA network.

Nevertheless, the relevance of lncRNAs and cytokines or macrophages in intraocular neovascularization is still unclear. Our GO analysis suggested that several altered lncRNAs are involved in the regulation of cytokines and immunological networks in the pathogenesis of CNV, however, further studies should be guaranteed to investigate the exact functions and mechanisms of the dysregulated lncRNAs. Chemokines are a group of small chemoattractant cytokines, which play great roles in inflammation and regulating angiogenesis as well as macrophage polarization 45, 46. Studies have shown that chemokine (C-X-C motif) ligand 8 (CXCL8) and monocyte chemoattractant protein-1 (MCP-1), and chemokine receptors such as CXC chemokine receptor 3 (CXCR3) have been reported to be involved in CNV and AMD pathogenesis 47, 48. A study showed that miR-539-5p attenuates experimental CNV through targeting CXC chemokine receptor 7 (CXCL7) 49, indicating that non-coding RNAs are involved in the functional effect of chemokines and their receptors that contribute to the formation of CNV. In the present study, GO and KEGG analyses showed that altered genes enriched in immune system process, immune response, chemokine signaling pathway and cytokine-cytokine receptor interaction, indicating that lncRNAs may regulate chemokines, cytokines and their receptors in the pathogenesis of CNV formation through their target genes. Thus, it is worth further investigate the mechanisms of involvement of lncRNAs in chemokines and chemokine receptors in CNV and AMD.

*Zhu et al.* presented identified lncRNAs involved in early AMD 21. In that study, GO and KEGG analyses showed that lncRNA-related genes enriched different functions, such as visual perception, sensory perception of light stimuli, and phototransduction pathway, but not immunological regulations. It might because that study focused on early-stage AMD patients without geographic atrophy and CNV, and our present study investigated the pathogenesis of CNV, which demonstrated different roles of lncRNAs in AMD.

We recently demonstrated that both mRNAs and lncRNAs have differential expression profiles in the oxygen-induced retinopathy (OIR) mouse model 20, which is used for investigation of retinal neovascular diseases. We recognized 539 altered mRNAs and 373 altered lncRNAs in OIR retinas (FC≥2.0, P<0.05) 20. And we tried to check the intersection of altered RNAs in both models, and identified 161 mRNAs and 53 lncRNAs which altered in OIR as well as CNV models, and the majority of the RNAs have the same trend of alteration. For instance, ENSMUST00000165968 and CD68 were up-regulated in both models, while ENSMUST00000144657 and cpa2 were down-regulated in both models. These double-dysregulated mRNAs and lncRNAs might play more essential roles in angiogenesis, and this suggested that lncRNAs could have potential roles in the pathogenesis of ocular neovascular diseases.

In conclusion, the present study reveals that lncRNAs and mRNAs are significantly altered in CNV mice through microarray analysis. Further, GO and pathway analyses indicate that altered lncRNAs are not only involved in biological processes of immune mechanisms and inflammation but also are involved in the related pathways which might contribute to CNV and AMD pathogenesis. In particular, immunological networks, including cytokines, chemokines and their receptors are mainly involved and relevant to the effect of lncRNAs in CNV. The limitation of this study is the lack of functional assessment of the identified lncRNA and mRNAs. Therefore, further studies are required to investigate the potential roles and exact mechanisms of the altered lncRNAs and mRNAs in CNV.

## Figures and Tables

**Table 1:** Sequence of the primers for lncRNAs.

	Forward and reverse primer	Tm (℃)	Product length (bp)
GAPDH (MOUSE)	F:5' CACTGAGCAAGAGAGGCCCTAT3' R:5' GCAGCGAACTTTATTGATGGTATT3'	60	144
uc009ewo.1	F:5' GGTAAGTCCCCACTATCATTCTC 3' R:5' AAACACCTTTGCCCTCCTC 3'	60	184
AK148935	F:5' TTTGTTGGCTGCCTTCTTTC 3' R:5' TGACTAACCTGTGAGTGTCCCTA 3'	60	72
uc029sdr.1	F:5' CTCTTGATGTATCCCAGGGTG 3' R:5' CAGGAAACACAATGCTACTCTC 3'	60	210
ENSMUST00000132340	F:5' CGGAATGTTACTGCCCATAG 3' R:5' TGGTCATTAGGATAAGTTCTGG 3'	60	254
AK030988	F:5' TGGGACCTAAGGATGGAAGA 3' R:5' AGACCAGAGCCAATGTGAGC 3'	60	167
uc007mds.1	F:5' TTCAGCCCCGACGAGCAC 3' R:5' GAAAGGGTTTGTTCGGCTCAC 3'	60	187
ENSMUST00000180519	F:5' TCTGGTTCTCGGCTATGTGC 3' R:5' AACAACAGCACAAACAGGGT 3'	60	129

Tm: temperature. bp: base pair.

**Table 2:** Top 20 significantly up-regulated lncRNAs.

Sequence Name	P-value	FDR	Fold Change	Regulation	Strand	Relationship	CNV 1	CNV 2	CNV 3	Control 1	Control 2	Control 3
AK036888	0.000171	0.150561	13.378940	up	-	intergenic	6.585418	6.480785	6.071276	3.077727	2.512265	2.321811
uc007pgi.1	0.014847	0.390976	9.578848	up	-	intergenic	5.211190	7.107776	4.430212	2.325983	2.321827	2.321811
AK078702	0.000536	0.150561	7.117427	up	-	intergenic	6.359641	6.132188	6.161450	3.862077	3.371296	2.925838
AK035526	0.005083	0.298875	6.657825	up	+	natural antisense	5.724644	7.089529	7.076339	4.231499	3.878298	3.575563
uc009ewo.1	0.000984	0.182037	5.719433	up	-	intron sense-overlapping	8.163315	7.829017	7.371524	5.300458	5.566779	4.949003
AK036625	0.005126	0.298875	5.353162	up	-	intergenic	5.726749	5.680111	5.143081	2.325983	3.345529	3.617254
ENSMUST00000148005	0.001561	0.218932	5.264145	up	+	exon sense-overlapping	6.509248	6.302205	5.601678	3.998450	3.479648	3.746437
ENSMUST00000153541	0.000164	0.150561	4.957192	up	+	exon sense-overlapping	11.456490	11.349358	10.999685	9.005207	9.098501	8.773256
AK008836	0.004393	0.293747	4.888444	up	-	intronic antisense	7.371524	6.772026	6.602773	4.573347	5.202004	4.102845
ENSMUST00000146254	0.000028	0.106609	4.640225	up	-	exon sense-overlapping	7.526886	7.218386	7.363228	5.129217	5.254380	5.082319
NR_026843	0.001265	0.203615	4.591574	up	-	natural antisense	6.264922	6.398417	5.991045	4.478630	3.930072	3.648715
ENSMUST00000122340	0.001105	0.191468	4.442445	up	-	intronic antisense	8.984519	8.526921	8.527271	6.376287	6.936308	6.272055
AK082144	0.000016	0.106609	4.424734	up	-	intergenic	6.554079	6.639836	6.746826	4.380675	4.513834	4.609461
AK048117	0.001609	0.221747	4.382340	up	-	intergenic	9.343423	9.431755	9.431267	7.627238	6.720520	7.463583
ENSMUST00000143423	0.000941	0.181475	4.307678	up	+	exon sense-overlapping	4.561347	5.009892	4.564078	2.963872	2.321827	2.528887
AK148935	0.000638	0.156222	4.263167	up	-	intergenic	8.259856	8.525405	8.333067	6.682964	6.086507	6.073080
AK047865	0.001065	0.191468	4.256438	up	-	intergenic	9.039629	8.963352	9.098866	7.275191	6.469285	7.088430
ENSMUST00000141975	0.000283	0.150561	4.232972	up	-	exon sense-overlapping	6.736901	6.384502	6.153523	4.416513	4.338800	4.274602
uc029sdr.1	0.000052	0.120829	4.185189	up	+	intergenic	10.768113	10.641598	10.589025	8.507404	8.798939	8.496515
NR_045384	0.010196	0.362008	4.172997	up	+	intergenic	5.724329	5.884554	5.893274	4.532355	3.801238	2.985313

P-values were calculated using unpaired t-test. FDR: false discovery rate. Fold change: the absolute ratio (no log scale) of average normalized intensities between CNV group and control group. CNV 1 to 3 and Control 1 to 3: each sample's normalized intensity (log2 scale). Hereinafter the same.

**Table 3:** Top 20 significantly down-regulated lncRNAs.

Sequence Name	P-value	FDR	Fold Change	Regulation	Strand	Relationship	CNV1	CNV2	CNV3	Control1	Control2	Control3
ENSMUST00000135495	0.044869	0.516189	7.465422	down	-	exon sense-overlapping	4.264994	6.259270	3.847529	8.927113	6.595256	7.550095
ENSMUST00000151832	0.042431	0.506906	3.682991	down	+	exon sense-overlapping	3.973213	5.239059	4.472576	5.519285	6.476424	7.331772
AK079094	0.000472	0.150561	2.875171	down	-	natural antisense	4.097720	3.991172	3.967711	5.716774	5.645347	5.265424
ENSMUST00000171338	0.003991	0.284707	2.831007	down	-	exon sense-overlapping	4.347292	4.591844	4.052868	6.017756	5.435850	6.042344
ENSMUST00000147716	0.002243	0.244886	2.805529	down	-	intergenic	7.029440	6.772935	7.329016	8.414517	8.813207	8.368486
AK030988	0.004724	0.293747	2.720887	down	-	intergenic	7.828629	8.353325	8.177764	9.318910	9.964228	9.408810
ENSMUST00000148202	0.005363	0.303718	2.572706	down	-	intergenic	3.028002	2.478576	3.041757	3.901149	4.463217	4.273829
AK005624	0.012396	0.380110	2.559854	down	+	bidirectional	4.166821	3.256077	3.443728	5.159213	5.085467	4.690131
AK009740	0.014086	0.383884	2.485166	down	-	bidirectional	2.530040	3.314212	2.545198	4.152919	4.397770	3.778787
ENSMUST00000155217	0.000964	0.182037	2.460863	down	-	intronic antisense	7.949919	8.236430	8.067902	9.477743	9.536148	9.137853
NR_037995	0.007062	0.335485	2.453643	down	-	bidirectional	7.028992	6.600705	6.301580	8.136388	8.017734	7.661932
ENSMUST00000155625	0.004510	0.293747	2.406930	down	-	exon sense-overlapping	2.323709	2.787010	2.712476	4.039022	4.044215	3.541541
uc007lit.1	0.039689	0.498925	2.389210	down	+	natural antisense	3.383898	2.440709	2.323922	3.760049	4.469326	3.688754
ENSMUST00000141135	0.027081	0.445563	2.334129	down	+	exon sense-overlapping	3.241041	2.759476	2.558381	3.748812	3.811546	4.667192
NR_033584	0.001977	0.240414	2.331771	down	+	intergenic	4.933888	5.203815	5.150325	6.451165	6.479938	6.021205
TCONS_00016118	0.007575	0.340233	2.312752	down	-	intronic antisense	5.561865	5.562532	4.940521	6.790447	6.549662	6.353641
AK029573	0.013265	0.380110	2.305416	down	+	natural antisense	4.500548	3.852448	3.562495	5.301173	5.106782	5.122617
ENSMUST00000144044	0.040861	0.503207	2.225878	down	+	exon sense-overlapping	4.548734	5.325275	4.294675	6.335644	5.575793	5.720369
NR_033469	0.005124	0.298875	2.225231	down	+	intergenic	6.507724	6.533446	6.704603	7.941327	7.926619	7.339692
AK013439	0.011666	0.378564	2.208164	down	+	intergenic	6.454914	6.874332	6.432539	7.343084	8.090536	7.756705

**Table 4:** Top 20 significantly up-regulated mRNAs.

Sequence Name	Gene Symbol	P-value	Fold Change	Regulation	Chrom	CNV 1	CNV 2	CNV 3	Control 1	Control 2	Control 3
NM_011784	Aplnr	0.000012	11.110603	up	chr2	7.033988	6.843315	7.167412	3.528313	3.702581	3.392226
NM_011646	Try4	0.001518	10.794919	up	chr6	5.868813	6.207469	6.427937	3.563739	2.321827	2.321811
NM_008372	Il7r	0.000099	9.886701	up	chr15	6.023879	5.297020	5.565189	2.325983	2.321827	2.321811
NM_177843	Gm14461	0.001185	8.779374	up	chr2	7.331272	7.067940	6.677774	3.347988	4.486817	3.839826
NM_001003405	Try5	0.000012	8.310638	up	chr6	5.221975	5.431142	5.596919	2.325983	2.437364	2.321811
NM_206535	Cd200r2	0.000381	7.767570	up	chr16	6.312686	6.182743	5.670891	2.734021	3.294071	3.265839
NM_175406	Atp6v0d2	0.001067	7.672187	up	chr4	10.914325	10.474780	10.348221	7.376840	8.241582	7.299991
NM_011089	Pira2	0.000091	7.671351	up	chr7	6.094800	5.933179	5.760588	2.792479	3.300848	2.876798
NM_207244	Cd200r4	0.006075	7.548943	up	chr16	6.464434	6.282340	5.594127	2.325983	3.989506	3.276587
NM_008311	Htr2b	0.000706	7.192641	up	chr1	10.557041	10.345577	9.884406	7.819662	7.395292	7.032505
NM_011088	Pira11	0.000196	6.976422	up	chr7	10.466558	10.217773	10.155017	7.762837	7.555972	7.113078
NM_011094	Pira7	0.000161	6.963833	up	chr7	10.961688	10.969189	10.845609	8.455120	8.155355	7.766367
NM_028973	Lrrc15	0.012774	6.827810	up	chr16	8.322155	8.931940	10.139378	6.901063	6.511550	5.666592
NM_178241	Cxcr1	0.003685	6.805119	up	chr1	6.005635	5.869263	5.788451	3.879585	2.321827	3.162076
NM_001166672	Gm14548	0.000097	6.673624	up	chr7	11.580840	11.296632	11.179657	8.847641	8.587764	8.406313
NM_019948	Clec4e	0.000563	6.596644	up	chr6	5.670682	5.027602	5.075513	2.887439	2.399350	2.321811
NM_011087	Pira1	0.003576	6.187779	up	chr7	5.488713	5.426346	5.204069	2.325983	3.583068	2.321811
NM_001267695	Ctss	0.000957	5.934620	up	chr3	9.404564	8.806512	8.751737	6.192754	6.833984	6.228608
NM_008848	Pira6	0.000255	4.851621	up	chr7	4.801415	4.635361	4.962917	2.325983	2.838959	2.399350
NM_008404	Itgb2	0.001909	4.841209	up	chr10	9.670052	8.907650	8.869934	6.768681	7.213496	6.639358

**Table 5:** Top 20 significantly down-regulated mRNAs.

Sequence Name	Gene Symbol	P-value	Fold Change	Regulation	Chrom	CNV 1	CNV 2	CNV 3	Control 1	Control 2	Control 3
NM_001024705	Prpmp5	0.014868	3.605863	down	chr6	9.067530	8.986222	9.954781	11.192267	11.751723	10.615577
NM_019840	Pde4b	0.001704	2.927001	down	chr4	3.256077	3.624609	3.076331	5.079052	4.632161	4.894074
NM_175296	Mael	0.013959	2.785800	down	chr1	5.162247	5.565056	5.125866	7.062294	7.111513	6.113637
NM_008623	Mpz	0.008683	2.769509	down	chr1	12.174806	13.137897	12.638795	13.881432	14.154913	14.324043
NM_183160	Tmem252	0.006698	2.677545	down	chr19	3.883402	4.178289	4.299822	5.111757	5.964037	5.548451
NM_207280	Ccdc121	0.014313	2.450424	down	chr1	2.805933	2.324675	2.323922	3.639443	3.399682	4.294497
NM_001025255	Mbp	0.000151	2.427744	down	chr18	2.949733	3.182399	3.054139	4.463824	4.263468	4.297829
NM_026925	Pnlip	0.028181	2.426672	down	chr19	3.116513	2.324675	2.323922	3.895614	3.380287	4.326145
NM_001113373	Shank2	0.023401	2.379186	down	chr7	3.648482	3.438832	2.672217	4.131874	4.666463	4.712598
NM_001081153	Unc13c	0.034934	2.372052	down	chr9	11.000760	10.198641	11.528724	12.222275	12.268342	11.975915
NM_001122594	Phlpp2	0.001196	2.356591	down	chr8	3.131635	2.660591	2.931842	4.163803	4.026565	4.243805
NM_001161722	Tfeb	0.003061	2.335554	down	chr17	2.690098	3.230295	2.904800	4.046248	4.066880	4.383359
NM_028807	Exoc3l4	0.026008	2.331869	down	chr12	4.272006	3.720309	3.146549	5.178742	4.693262	4.931320
NM_001127685	BC048943	0.013403	2.324064	down	chr12	9.447387	8.766418	8.507593	10.125555	10.236406	10.009386
NM_001204253	Clec1b	0.008321	2.321635	down	chr6	4.644223	5.290836	4.979474	5.859919	6.408708	6.291329
NM_007545	Hrk	0.003712	2.290870	down	chr5	7.678339	8.078760	7.425854	8.965889	8.983374	8.821377
NM_010220	Fkbp5	0.029949	2.281321	down	chr17	6.708265	6.429901	7.553089	8.247108	8.176554	7.837201
NM_020599	Rlbp1	0.007127	2.235286	down	chr7	8.194874	8.097004	7.941646	9.260371	9.602041	8.852490
NM_147110	Olfr570	0.003044	2.223418	down	chr7	5.491363	5.307785	5.178571	6.613100	6.653939	6.169017
NM_027790	Dhrs2	0.003418	2.203722	down	chr14	5.776111	6.199949	5.806094	6.968227	7.311111	6.922644
